# Clinical Implementation of Rare and Novel* DPYD* Variants for Personalizing Fluoropyrimidine Treatment: Challenges and Opportunities

**DOI:** 10.7150/ijbs.97686

**Published:** 2024-07-02

**Authors:** Elena De Mattia, Noemi Milan, Yehuda G. Assaraf, Giuseppe Toffoli, Erika Cecchin

**Affiliations:** 1 Experimental and Clinical Pharmacology, Centro di Riferimento Oncologico di Aviano (CRO) IRCCS, via Franco Gallini n. 2, 33081 Aviano (PN), Italy.; 2 The Fred Wyszkowski Cancer Research Laboratory, Faculty of Biology, Technion-Israel Institute of Technology, Haifa 3200003, Israel.

**Keywords:** *DPYD*, rare variant, fluoropyrimidine, toxicity, next-generation sequencing, clinical implementation

## Abstract

Fluoropyrimidines (FLs) [5-Fluorouracil, Capecitabine] are used in the treatment of several solid tumors. Dihydropyrimidine dehydrogenase (DPD) is the rate-limiting enzyme for FL detoxification, and its deficiency could lead to severe, life-threatening or fatal toxicity after FL administration. Testing with a pharmacogenetic panel of four deleterious variants in the dihydropyrimidine dehydrogenase gene *(DPYD)* (DPYD*2A, DPYD*13, c.2846A > T, c.1129-5923C > G) prior to FL treatment, is recommended by scientific consortia (e.g., CPIC, DPWG) and drug regulatory agencies (e.g., EMA). However, this panel identifies < 20% of patients at risk of severe FL-related toxicity. Cumulative recent evidence highlights the potential clinical value of rare (minor allele frequency < 1%) and novel *DPYD* genetic variants for identifying an additional fraction of DPD-deficient patients at increased risk of severe FL-related toxicity. In this review, we aimed to comprehensively describe the available evidence regarding the potential clinical predictive role of novel and rare *DPYD* variants as toxicity markers in FL-treated patients, and to discuss the challenges and opportunities in tailoring FL treatment based upon clinical application of such markers. Although we must overcome existing barriers to the clinical implementation, the available data support that comprehensive assessment of the *DPYD* sequence, including rare and novel genetic variants, may significantly enhance the pre-emptive identification of at-risk patients, compared to the current targeted approach.

## Fluoropyrimidine-Related Adverse Drug Reactions and *DPYD* Testing

Since the 1950s, 5-fluorouracil (5-FU) has been one of the most commonly prescribed anticancer drugs for the treatment of various solid tumors 1, 2. Belonging to the antimetabolite fluoropyrimidine (FL) class, 5-FU and its oral prodrug capecitabine act as false high-affinity substrates for thymidylate synthase, thereby inhibiting pyrimidine biosynthesis in cells displaying high proliferation rates. Metabolites of 5-FU exhibit potent cytotoxic activity due to the biosynthetic depletion of endogenous thymidine, along with direct damage to DNA and RNA, leading to cell death and tumor growth suppression. While the pharmacological efficacy of FLs is well-established, the narrow therapeutic index is a major issue in the management of chemotherapy. Although the vast majority of patients can be safely treated with FLs, 20-30% will develop severe or even life-threatening untoward toxicity during the course of chemotherapy, resulting in treatment delays and patient discomfort 3.

Inter-individual differences in the pharmacokinetics and pharmacodynamics pathways of FLs could play a role in the observed variability in therapeutic outcome. The metabolic pathway of FLs includes a number of proteins such as ATP-binding cassette (ABC), solute carrier transporters (SLC), nuclear receptors, and enzymes (e.g., thymidine phosphorylase, uridine monophosphate synthase, cytidine deaminase), that have been previously reported to have genetic polymorphisms which could affect drug bioavailability and exposure to some extent. Moreover, thymidylate synthase, which is the drug target, and 5,10-methylenetetrahydrofolate reductase, which is involved in the pharmacodynamic effect of the drug, have been reported to play a relevant pharmacogenetic role in the toxicity and efficacy of FLs 4-6.

One more specific cause of FL-related toxicity is inefficient catabolism of the drug, which is mainly mediated by the detoxification enzyme dihydropyrimidine dehydrogenase (DPD). Many efforts have been made to better characterize the genetic basis of this metabolic defect 7-9. Currently, only four single-nucleotide polymorphisms in the dihydropyrimidine dehydrogenase gene (*DPYD*) are classified as clinically relevant, and listed in international pharmacogenetics guidelines for drug dose recommendations—such as those provided by the Clinical Pharmacogenetics Implementation Consortium (CPIC) 10 and the Royal Dutch Association for the Advancement of Pharmacy (DPWG) 11. The following variants are known for their role in impairing DPD activity: DPYD*2A (rs3918290) and DPYD*13 (rs55886062), which are associated with nearly complete protein deficiency in homozygotes 12, and c.2846A>T (rs67376798) and c.1129-5923C>G (rs75017182, tagging HapB3), which are associated with moderate loss of protein function 7. On May 2020, this compelling evidence prompted the European regulatory agency to publish its own pharmacogenetic recommendations to improve appropriate FL use. EMA now recommends using a reduced initial dose of FLs in patients with DPD deficiency, as determined either by phenotyping (i.e., measuring plasma uracil concentration) or by genotyping patients with the four-variant *DPYD* panel 13.

Notably, the four-variant *DPYD* panel presents a high specificity (between 99% and 100%) but a low sensitivity (1-12%) for detecting patients at risk of toxicity 8, suggesting that other factors—genetic or otherwise—are potentially involved in the development of FL toxicity, and indicating a need for further investigation of the *DPYD* genotype. It also should be noted that the 4-variant test presents a high specificity, with carriers of these four alleles presenting a high risk for severe toxicity, and a low sensitivity with non-carriers still presenting a high risk of unpredictable toxicity occurrence 14. Considering the reported complexity of *DPYD* genetics, it is likely that many additional rare genetic variants may contribute to the observed interindividual variability in the risk of toxicity development 15. Moreover, about 7% of Europeans are carriers of at least one of the four variants included in the international guidelines, while some of these variants are very rare in populations of African or Asian origin, where additional variants might play a more relevant role 10.

In the present article, we systematically review the available literature regarding the role of genetic *DPYD* variants in identifying patients at high risk of toxicity following FL administration. In addition to the four currently recommended variants, we also focused on variants that are defined as “rare” in the studied population [minor allele frequency (MAF) < 1%] or that have never been previously reported. We also discuss the potential hurdles for the clinical implementation of using these rare and novel variants as markers untoward toxicity.

## Methods

We performed a systematic literature search for all published studies addressing the role of genetic germline *DPYD* variants that were classified by the authors as “rare” or “novel”, and that were potentially related to severe (grade 3-5) or lethal toxic reactions to FL-based treatment in cancer patients. In general, “rare” was defined as a variant not classifiable as a common polymorphism, and “novel” was defined as a variant absent from the available public databases (i.e., NCBI SNP database, Ensembl) 16, 17 at the time of study publication. For each identified study, we recorded information related to patients' status regarding the routinely tested *DPYD* markers (DPYD*2A, *13, c.2846A > T, c.1129-5923C >G-HapB3). This systematic review was carried out according to Preferred Reporting Items for Systematic Reviews and Meta-Analyses (PRISMA) guidelines, and the protocol was registered in the International Prospective Register of Systematic Reviews (PROSPERO 2024 CRD42024501461 Available from: https://www.crd.york.ac.uk/prospero/display_record.php?ID=CRD42024501461).

Three databases—MEDLINE (PubMed), Cochrane Library, and Web of Science Core Collection (Clarivate)—were searched for relevant articles, with the last search update on March 1, 2024. Since MEDLINE included all of the articles returned by the other two databases, we referred only to MEDLINE. Search algorithms included the keywords “*DPYD”*, “rare variant”, “fluoropyrimidine (5-fluorouracil or capecitabine)”, and “toxicity”, combined with Boolean operators (OR/AND). Additional studies were identified by manually searching the references of relevant articles. To evaluate the studies retrieved using this search strategy, two independent authors (EDM and EC) screened the titles and/or abstracts, and selected suitable studies according to the inclusion and exclusion criteria. Then, full-text versions of these potentially eligible articles were retrieved and independently assessed by these two authors. Disagreements were settled by a third researcher (GT). When studies overlapped, the publication with the largest number of patients was included in this systematic review.

**Figure 1:**
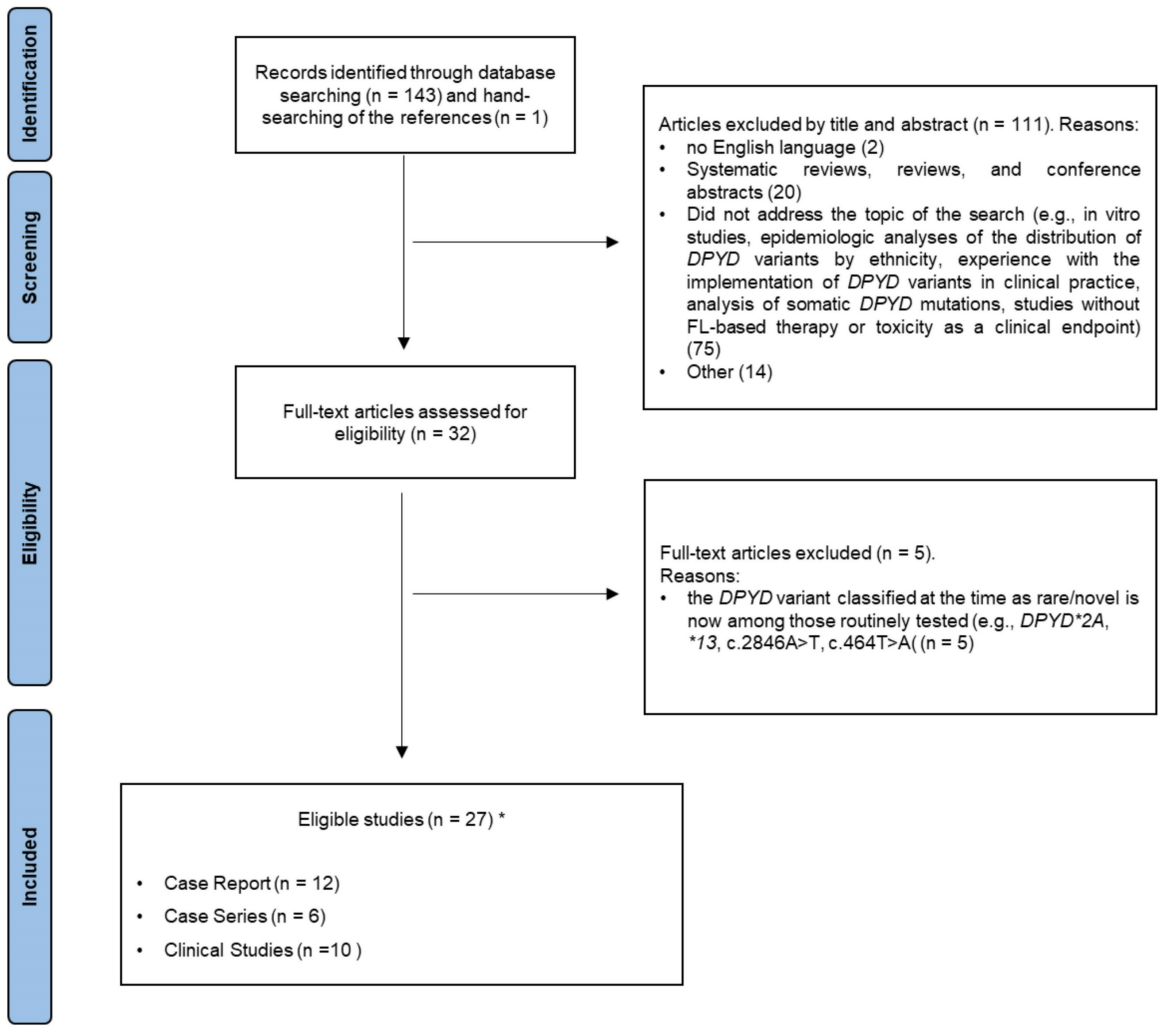
** PRISMA flow diagram.** *One study was both a case report and a clinical study.

Inclusion criteria were that the studies were published in English in a peer-reviewed journal, and contained data regarding the topic of the present review. Given the qualitative nature of this work, we included case reports, case series, and descriptive analyses. Systematic reviews, reviews, and conference abstracts were excluded. Similarly, we excluded papers that did not address the topic of the search—e.g.,* in vitro* studies, epidemiologic analyses of the distribution of *DPYD* variants by ethnicity, experiences with implementation of *DPYD* variants in clinical practice, analysis of somatic *DPYD* mutations, and studies without FL-based therapy or toxicity as a clinical endpoint. Figure 1 shows the flowchart of our literature search. Ultimately, a total of 27 studies were included and discussed in the present systematic review.

For each eligible study, the following items were recorded in a pre-piloted form: the country where the study was based; the analytical method used to detect *DPYD* variants; the clinical description of cases (for case reports and case series) or the patient population (for clinical studies), including data about the cancer type and patient sex/gender; therapy characteristics (e.g., 5-FU or capecitabine administration, monotherapy or combined therapy, and clinical setting); strategies used for the functional prediction of detected variants; method of DPD phenotyping; and a summary of the main findings of the article. Data related to the patients' status for the routinely tested *DPYD* markers was recorded when available (Table 1 and Table 2).

Since the present work is a review of qualitative studies, the risk of bias quality assessment is not applicable.

## *DPYD* Genetic Complexity and the Role of Rare Genetic Variants

Compared to conventional targeted genotyping strategies, recently developed high-throughput next-generation sequencing (NGS) technologies have provided a more complete picture of the variability of candidate genes by elucidating the full spectrum of variants, including rare ones (MAF < 1%). The Exome Aggregation Consortium has reviewed data from international population-scale sequencing programs (e.g., 1000 Genomes Program), revealing that most human germline variants are rare or novel 18 and that the probability of a variant being deleterious is inversely related to its frequency 19. Therefore, focusing on rare variants could increase the likelihood of finding functionally deleterious and impactful ones.

A growing body of recently published data shows that rare and novel variants may also have significant clinical value for precision medicine 20-24. In particular, a series of studies investigated the genetic variability of clinically relevant genes (i.e., transporters, phase I and II enzymes, and nuclear receptors), and demonstrated that approximatively 30-40% of the overall functional variability of these genes is caused by rare variants that are not commonly captured by targeted genotyping approaches 25-27. These same studies also revealed that rare pharmacogenetic variants are highly enriched in mutations predicted to be functionally relevant, and that these rare variants are responsible for much of the unexplained inter-individual variability in drug metabolism phenotypes, pharmacokinetics, and risk of adverse drug reactions.

A recent study compared sequencing-based and targeted genotyping-based approaches and demonstrated that, among pharmacogenes, *DPYD* had the largest number of unique potentially clinically relevant variants that were missed by standard targeted genotyping 28. The sequencing design adopted in this analysis captures the genetic variability of all exons, splice junctions, upstream and downstream regulatory regions for each gene as well as also includes probes corresponding to additional sites present for several commercial pharmacogenetic microarray platforms. The study found that approximately 6% of the patient cohort (n = 631/10,030) carried a *DPYD* variant that was missed by the *in silico* targeted genotyping panel, and over 100 additional* DPYD* variants were identified by sequencing with respect to the targeted-approach, each found in only one or a few patients 28. The further analysis of Zhou et al., revealed that, unlike other pharmacogenes (e.g., *TPMT*), a very high number of variants must be interrogated for *DPYD* gene in order to explain the functional variability based on *in vitro* and* in vivo* data 15. For instance, 421 *DPYD* variants need to be interrogated to explain 99% of the DPD deficiency 15. Notably, the four routinely tested *DPYD* variants identified only a minimal percentage of the genetically determined DPD deficiency, and approximately 17% of FL-related severe toxicity. These findings have important implications for genotype-guided prescribing, demonstrating that comprehensive sequencing of the *DPYD* gene could improve the identification of genetic variants that cause FL toxicity.

## Case Reports and Case Series

Our search identified a number of published case reports and case series 29-46 that examined the potential genetic causes of FL-related extreme adverse reactions, including death. Most of these investigations applied a retrospective direct sequencing approach to identify previously uninvestigated variants in the *DPYD* gene that could be responsible for the observed severe toxic reactions (Table 1). In most studies, the sequencing included all exons and in some cases the adjacent intronic region as well, while a broader design and sequencing of the whole gene was only performed in a few studies. The papers described single or a few cases from clinical practice, mainly involving patients with gastrointestinal or breast cancer, who received FLs (i.e., 5-FU or capecitabine) alone or in combination with other drugs, and who experienced early-onset grade ≥3 or even lethal toxicity. Five out of twelve case reports described lethal toxicity cases. Comprehensive *DPYD* sequencing was often performed because the patients were found to have a constitutive deficiency in DPD activity that was not related to, or not completely explained by, the four routinely tested *DPYD* variants. In most cases, the analysis revealed some genetic variants in *DPYD* that the authors classified as “rare” or “novel” variants, and which were hypothesized to explain the observed adverse effects.

Defining the functional impact of the detected *DPYD* variants is a critical step towards determining whether they caused an observed toxic side effect. In most of the cases reviewed herein, the identified variants were classified as “functionally relevant” based on different approaches, including *in silico* tools, *in vitro* and molecular analyses, crystal DPD structure analysis, and literature search. Interestingly, in one case, the identification of a rare *DPYD* variation in one patient helped to personalize the FL-based therapy for another family member diagnosed with cancer, thus preventing potential severe toxic effects 29, 30. In another case, the extreme toxic event described did not appear to be related to a single variant, but rather to a rare haplotype combination of more frequent *DPYD* variants: c.85T>C + c.496A>G + c.1601G>A + c.1627A>G or c.496A>G + c.1601G>A 35. Table 1 presents the outcomes of the toxic events, either resulting in treatment interruption or dose adjustment.

### Case-control Clinical Studies

Several single case reports or small case series identified previously uninvestigated *DPYD* variants as potential causative markers of extreme FL-related toxicity, thereby encouraging further investigation on a larger scale. In this context, a number of case control studies were conducted to explore the possibility of performing comprehensive genetic analysis of *DPYD* to highlight “rare” or “novel” variants as risk factors for severe toxicity after FL-based treatment 21, 43, 47-54 (Table 2). Four studies 43, 49, 51, 53 reported the functional characterization of several *DPYD* variants—including a number of rare and novel variants—that were identified by sequencing all exons and flanking intronic regions of *DPYD* gene using the Sanger method in a group of patients with DPD deficiency, who developed early-onset severe toxicity. One of these studies 49 also reported that 90% of the screened patients (25/28) had at least one variant in the *DPYD* coding sequence, about half of which were potentially deleterious based on functional prediction performed using multiple *in silico* tools and protein structure modeling. In another investigation 50, the coding* DPYD* region was sequenced in 968 cancer patients using the NGS method. It was found that one of the patients had a very rare *DPYD* missense variant (c.G1651G>A; [p.Ala551Thr]), which was predicted to be strongly deleterious by *in silico* tools and literature review, and was considered to have caused the observed grade 4 hematological toxicity episode.

Three more recent studies 47, 48, 52 have attempted to quantify the increased percentage of toxicity cases that are explained by full *DPYD* exon sequencing, compared to the genotyping of the recommended four-variant panel. In a study of 94 cancer patients, De Luca et al., 47 showed that the detection of rare *DPYD* variants (MAF < 0.05) by exon sequencing resulted in a 2.5% improvement in the identification of patients with DPD deficiency, compared to screening only the four recommended *DPYD* variants, which identified 20% of DPD-deficient patients. In a smaller investigation of 33 cancer patients, Soria-Chacartegui et al., 52 applied* DPYD* coding region sequencing, and discovered the functionally relevant rare variants rs367619008 (c.187A>G, [p.Lys63Glu]) and rs200643089 (c.2324T>G, [p.Leu775Trp]), together with the more frequent variant rs76387818 (c.1084G>A, [p.Val362Ile]). The functional prediction was performed using *in silico* tools and literature review. These authors highlighted a larger increase in the identification of patients with toxicity—from 20-30% when using only the four recommended markers, to 38-48% with the additional identified variants. In another study of 243 advanced breast cancer patients receiving capecitabine, Etienne-Grimaldi et al., 48 investigated the value of integrating the three routinely tested variants according to the French pharmacogenetic guidelines (*2A, *13, and c.2846A>T) with an additional four rare variants having an *in vitro* verified deleterious effect (c.1475C>T, c.1774C>T, c.1025A>G, and c.300C>A) detected by direct sequencing of all exons plus flanking intronic part and the 3'/5' untranslated region (UTR) of* DPYD* gene. This approach improves the performance of genotyping for identifying patients developing grade 3-4 toxicities (sensitivity: 26.7%; PPV: 72.7%; RR: 7.6; *P* < 0.001) or only grade 4 toxicities (sensitivity: 60%; PPV: 27.3%; RR: 31.4; *P* = 0.001).

In two more recent case-control studies 21, 54, researchers have tried to statistically estimate the power of rare and novel variants to predict FL-related toxicity risk. In a study of about 200 Caucasian cancer patients treated with FL-based therapy, De Mattia et al., 21 demonstrated that the burden of rare and novel *DPYD* variants is significantly higher among patients with toxicity, compared to patients without toxicity by sequencing all exons, splice junctions, 3'/5' UTR and proximal promoter region of the* DPYD* gene. Specifically, among carriers of at least one rare missense *DPYD* variant, the risk of developing grade 3 toxicity or greater was 16-fold higher during the first treatment cycle (*P* = 0.013), and 11-fold higher throughout the course of chemotherapy (*P* = 0.025). Yokoi et al., 54 obtained similar results in a study including 301 Japanese cancer patients who received 5-FU alone or in combination with other drugs and sequencing the coding exons and flanking intron regions of the *DPYD* gene. Their analysis revealed that patients carrying at least one rare *DPYD* variant (MAF < 0.01) related to an *in silico* loss of function exhibited an increased risk of developing grade 3 toxicity or greater during the first two treatment cycles (*P* = 0.003).

### Analysis of the Identified *DPYD* Rare and Novel Variants

Table 3 presents detailed descriptions of each rare and novel *DPYD* variant that was highlighted by the herein reviewed case reports, case series, and clinical studies, and that was potentially associated with severe or lethal FL-related toxicity. Notably, the listed classification of these variants as “rare”, “very rare”, or “novel” (i.e., absent from available public database) are as provided by the authors of papers, and based on the available information at the time of paper publication. These classifications have been revised based on the data currently available in public databases, such as dbsnp 17 or Ensembl 16. A total of 46 germline aberrations were identified in the *DPYD* gene, of which, 24/46 (52.2%) were classified by the authors as novel, and 21/46 (45.7%) as rare or very rare; one variant was not classified. Of the 24 novel variants, 10 were assigned an rs ID after study publication. Most of the identified variants were missense (32/46, 69.6%). The remaining variants included stop-gain variants (5/46, 10.9%), a deletion (1/46, 2.2%), an intronic variant (1/46, 2.2%), splice site variants (5/46, 10.9%), a variant within the 3' region (1/46, 2.2%) and a exons amplification (1/46, 2.2%).

Interestingly, five missense variations (c.257C>T [p.Pro86Leu], c.623G>A [p.Arg208Gln], c.775A>G [p.Lys259Glu], c.1601G>A [p.Ser534Asn, DPYD*4], and c.2324T>G [p.Leu775Trp]) were highlighted by more than one study, supporting their potential functional effects and roles in predicting the risk of severe toxicity, which warrants further investigation. One of these variants, c.1601G>A, has already been extensively investigated and is most common in the European population with respect to other ethnic groups 55. Notably, this variant has been linked to altered DPD activity and FL-associated toxicity, but the available evidence regarding its clinical validity has not yet been confirmed. A meta-analysis published in 2015 did not confirm a significant association between the c.1601G>A variant and severe FL-related toxicity; however, all included studies reported that variant carriers had a relative risk of toxicity over 1.0, suggesting some effect on toxicity 56. The most recently published papers have presented controversial results regarding the potential utility of implementing c.1601G>A as a marker in clinical practice, thus leaving an open question 55, 57, 58.

### Approaches for Functional Prediction of *DPYD* Genetic Variants

One challenge of using sequence-level data in the context of clinical implementation is the need to reliably and rapidly classify the identified novel and rare *DPYD* variants based on their functional impact on enzyme activity. Several strategies have been utilized in the above-discussed studies (Table 1), including *in silico* tool prediction, literature search, analysis of crystal DPD structure, and various kinds of molecular analyses (e.g., cDNA sequencing and *in vitro* functional assay). Although experimental assays in recombinant cell lines represent the gold standard for functional evaluation of pharmacogenetic variants, these methods are low-throughput, costly, time-consuming, and require trained laboratory staff, which render them unsuitable for point-of-care use 59, 60. Recently developed high-throughput experimental functional assays—such as deep mutational scanning to analyze protein-coding mutations—are not yet suitable for prompt functional prediction 60. Moreover, epidemiological analyses require adequately large sample sizes to achieve statistically significant results, which is not easily achievable for rare variants 59, 60.

Computational predictions appear to be the most suitable method for timely assessment of the phenotypic effects of rare and novel variants. A multitude of algorithms are available to evaluate the deleterious impact of a given variant based upon its sequence conservation, structural data, physicochemical properties of amino acid substitutions, and functional genomics information 61, 62. However, there exists a need for algorithms specifically designed for evaluating pharmacogenetic variants, since commonly used algorithms generally underperform when assessing variants of pharmacogenetic relevance 61, 62.

Machine-learning based approaches have enabled advances in this field. Specifically, a *DPYD*-specific variant classifier (*DPYD*-Varifier) has been trained on 156 missense *DPYD* variants with matched DPD activity *in vitro* data 63, achieving 85% prediction accuracy on a set of novel missense variants, hence outperforming other widely used *in silico* prediction tools, such as PROVEAN, SIFT, and PolyPhen-2. Zhou et al., recently developed a quantitative ensemble classifier—the ADME-optimized prediction framework (APF) algorithm—which is intended to assign the deleteriousness of missense variants in ADME-related pharmacogenes, including *DPYD*. This novel tool was trained exclusively on experimentally characterized pharmacogenetic variants. Compared to *in vitro* tests, it reportedly achieved a sensitivity and specificity of 93% for predicting loss-of-function and functionally neutral pharmacogenomic variants, outperforming conventional *in silico* tools, such as SIFT, Polyphen-2, PROVEAN, and CADD 15, 64. Notably, APF also performs very well on *DPYD* variants, even though no *DPYD* data were used for model training 59, 60. Interestingly, APF and *DPYD*-Varifier have shown an overall good agreement for the prediction of population-specific frequencies of DPD metabolizer phenotypes 15. However, additional efforts are required to further improve these novel *in silico* algorithms, and to overcome their current limits. Significant advances may derive from the use of large-scale training data sets, e.g., large-scale experimental mutagenesis screening, increased quantities of systematically collected large-scale functional evidence, and the possibility of testing on population-scale genomic biobanks with correlated electronic medical records. The functional classification of missense variants could also potentially be improved by the recently developed artificial intelligence-based structural prediction tools (e.g., AlphaMissense) 65, which open promising new opportunities. Despite progress in the functional classification of missense variants, the available *in silico* prediction tools for pharmacogenes remain to underperform for non-missense variants, e.g., for synonymous, nonsense, frameshift, splice, and non-coding variants 61, 62. Although efforts are being made to improve these tools, much work needs to be conducted in order to enable their implementation in a clinical context.

### Challenges for Clinical Implementation of an NGS Approach for FL Precision Dosing

**Figure 2:**
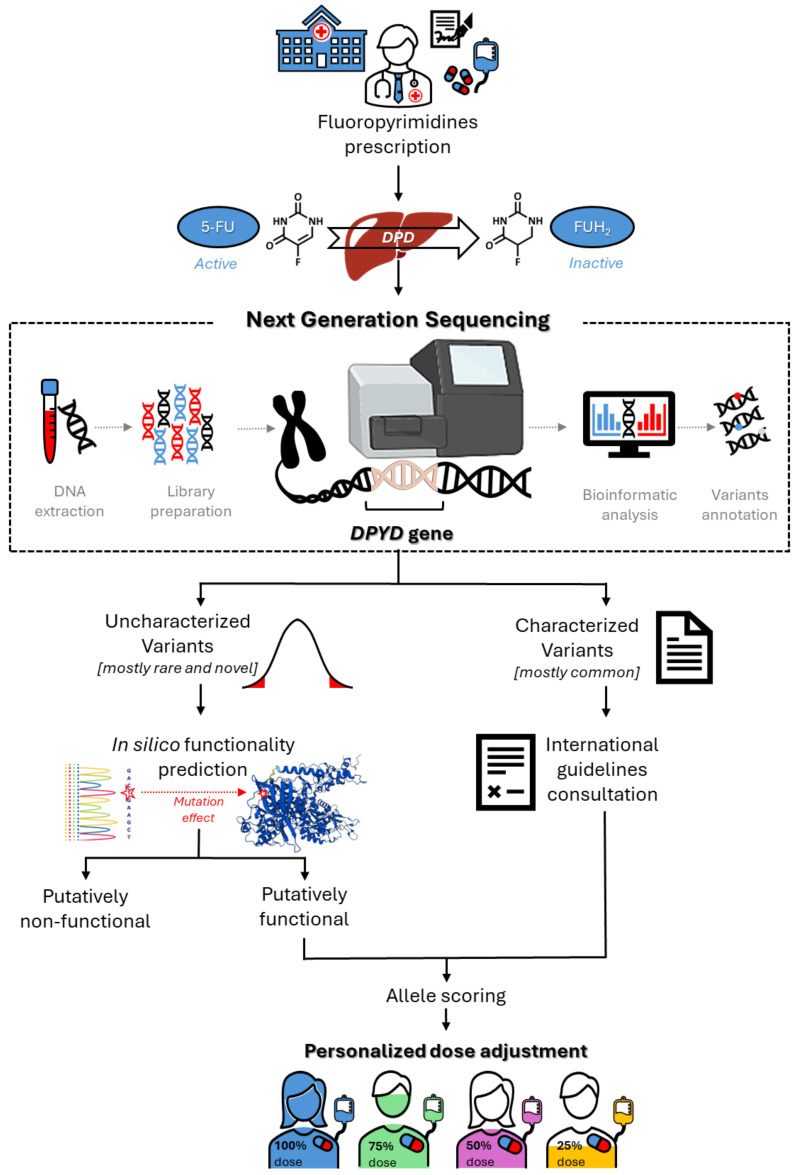
Roadmap for the clinical implementation of *DPYD* sequencing for optimization of fluoropyrimidine dose adjustment.

Considering the potential value of rare and novel *DPYD* variants for predicting FL-related toxicity risk, one major opportunity for the near future will be the translation of that information into clinical practice, to enable personalized therapy (Figure 2). However, some critical issues persist, and represent a challenge for pharmacogenetic research. Notably, the currently available data, although promising, are mainly derived from case reports or case-control studies with limited sample sizes, and remain to be validated in sufficiently powered prospective clinical studies (Tables 1 and 2). This will certainly require envisioning new models of prospective clinical pharmacogenetic trials, with the aim of pin-pointing the impact of a genetic variant based on only its minor allele frequency and its predicted functional effect. Clinical translation will also require concentrated efforts by scientific pharmacogenomic consortia to translate the evidence into clinical guidelines, with dose adjustment recommendations for carriers of rare and novel *DPYD* variants. In this context, there exists a need to develop reliable bioinformatic tools that enable quantitative functional prediction of rare and novel uncharacterized *DPYD* variants, as discussed in the previous section.

The implementation of rare *DPYD* variants as predictive biomarkers in clinical practice will also require formal assessment of the cost-effectiveness of sequencing-based analytical approaches, compared to targeted genetic analysis. It would be also worth considering the cost-effectiveness of the required infrastructural investments (e.g., laboratories with state-of-the-art equipment, and expertise) for timely and reliable genotype analyses. However, considering the increasingly widespread use of NGS technology, with associated lowering of costs and increased presence of equipment in laboratories, the adoption of *DPYD*-sequencing as routine clinical practice seems to be a realistic scenario. Regarding the turnaround time, NGS-based testing workflows (i.e., DNA extraction, library preparation, sequencing, data analysis, and reporting) typically require a few weeks 66, which limits their use for preemptive guidance of personalized prescribing in acute cases. However, a recent study reported that this turnaround time could be reduced to three business days by using a novel automated NGS assay, making the clinical application of NGS methods more realistic in the near future 67. Notably, smaller laboratories may require a longer time due to the need to obtain a minimum number of samples to perform NGS analysis. An additional shortcoming that must be addressed before NGS-based tests are introduced into clinical practice is the need for a more comprehensive analysis of analytical performance—at least including the performance characteristics recommended by international agencies (e.g., least accuracy, precision, limit of detection, specificity)—and the availability of regulatory guides for standardizing reports on the analytical validation of NGS approaches 68.

## Discussion

Overall, the published data reveal that standard screening of routinely tested *DPYD* variants is sometimes insufficient to prevent severe or fatal toxicity of FLs. Evidence suggests that more comprehensive *DPYD* sequencing approaches may potentially be useful for detecting rare and novel risk genetic variants, identifying a greater proportion of patients with DPD deficiency, who are likely at risk of developing adverse events related to FL treatment.

The preliminary published reports highlight the advantage that a sequencing-based approach provides a complete picture of the variability of the gene of interest (i.e., *DPYD*), thereby reducing bias attributable to test design, compared to a targeted genotyping-based test. It should be also noted that a sequencing-based approach allows for future re-analysis of the data using the most recent pharmacogenetic information that was not available at the time of the initial test. The published clinical cases also suggest that extensive *DPYD* sequencing could help to overcome the ethnic-specific distribution of *DPYD* variants described by some studies 15, 39, 46. Notably, testing by targeted genotyping may miss rare but potentially clinically relevant variants, and this is particularly evident among populations that have been historically underrepresented in research and genetic sequencing cohorts. For example, of the four *DPYD* variants that are validated for pre-treatment dose adjustment in patients of European descent, none has yet been identified in an Eastern Asian population 54, 69. Moreover, these four *DPYD* genetic markers present a low frequency among patients of African ancestry, where other variants may play an important role 70, 71. Extensive *DPYD* sequencing could be beneficial for detecting potentially relevant variants in different ethnic contexts, leading to both improved and more inclusive patient care 15, 30, 39, 46.

It should be highlighted that in addition to the *DPYD* variants identified in published studies that may be associated with FL-related toxicity (Table 3), other genetic *DPYD* alterations and rearrangements detected by gene sequencing have been described and reported as the cause of DPD deficiency and related pathologic syndromes 72-75. These molecular *DPYD* variations should be further investigated for their potential clinical role in predicting the risk of developing severe toxicity after FLs administration. The most promising among them is a *DPYD* exon 4 deletion detected by gene screening in the Finnish population and associated with DPD deficiency and preliminary FL-related toxicity, emphasizing that the potential value of *DPYD* copy number variation is currently underestimated 73, 76. It is presently unclear how variants' rearrangement in the haplotype structure might impact the phenotypic effect on DPD activity. Recent data demonstrate how variants with controversial phenotypic effects present significant levels of linkage disequilibrium (LD), and could be combined in haplotypes, allowing the stratification of patients according to both DPD activity 77 and risk of FL-related adverse drug reaction 78. In addition, advances in the NGS technology could be helpful in the definition of haplotype structures. Although previous research indicated that the two variants linked to the HapB3 haplotype, c.1129-5923C>G and c.1236G>A, are in perfect LD, a recent paper has highlighted rare cases of incomplete linkage within the three variants. This finding could raise a question as to the current *DPYD* clinical testing strategies that use c.1236G>A as a surrogate for the causative variant c.1129-5923C>G 79. This adds an additional level of complexity that must be addressed by future studies.

Considering that the variability in DPD enzyme activity can only be partly explained by genetic variants, several DPD-phenotyping methods have been implemented in clinical practice to identify more patients with DPD deficiency 80. In general, DPD phenotyping has shown higher sensitivity compared to *DPYD* genotyping, but prospective studies are still required to better define this aspect 80, 81. The gold standard for DPD phenotyping is the determination of DPD enzyme activity in peripheral blood mononuclear cells. As alternatives, several methods have been developed based upon analyzing the concentrations of dihydrouracil (UH_2_) and uracil (U), and their ratio in a patient's plasma, as surrogate markers for intracellular DPD activity. However, these tests have limitations, including a lack of consensus regarding the threshold of U concentration or UH_2_/U ratio to determine DPD deficiency, the requirement for specific equipment that is not readily available at all hospitals, and the fact that the results are strongly influenced by the blood collection time (e.g., U is influenced by circadian rhythm and food) and sample processing (e.g., the sample must be processed immediately due to the limited stability of U and UH_2_). These issues complicate the implementation of this DPD-phenotyping method in clinical practice, reinforcing the need for further research efforts to improve the guiding of FL dosing according to *DPYD* genetic testing.

Given the complexity of the metabolic pathway of FLs—with DPD and several other enzymes and metabolic proteins involved in determining drug bioavailability and exposure—a polygenic approach based on rare and novel variants should also be considered. In our recent case-control study 82, we demonstrated that the burden of rare dihydropyrimidinase (*DPYS*) variants was significantly higher in patients with toxicity compared to controls, and that the presence of at least one rare *DPYS* variant was associated with an approximately four-fold higher risk of severe toxicity. These data demonstrated that the rare mutational burden of *DPYS*—a gene that cooperates closely with *DPYD* in the catabolic pathway of FLs—is another promising pharmacogenetic marker for precision dosing of FL, which may improve treatment personalization, especially in cancer patients with normal DPD activity. Overall, further pharmacogenetic analyses should be performed to investigate a combined algorithm based on the burden of rare variants in multiple FL-related genes.

Another point to consider is that, in addition to genetic factors, non-genetic factors could also influence the risk of developing FL-related toxicity and should be taken into account as covariates. For example, a recent meta-analysis 83 has shown that some clinical variables such as gender, age, body mass index, administration schedule of FL and associated chemotherapeutic agents have significant predictive value. In particular, an increased risk of toxicity was observed in women, which is consistent with the lower lymphocytic DPD enzyme activity and 5-FU clearance described in this gender. Aging, low body surface area, use of 5-FU bolus and concomitant administration of other anticancer drugs were also associated with higher FL-related toxicity. In conclusion, although targeted genotyping detects the most clinically significant *DPYD* variants, sequencing-based approaches also enable the detection of potentially deleterious rare and novel *DPYD* variants that collectively affect many patients, and could predispose the patient to severe FL-related toxicity. Moreover, the adoption of sequencing-based strategies could enable more accurate patient metabolizer phenotype classification in historically understudied populations, where targeted genotyping may miss clinically relevant variants. Once the current critical issues are overcome, NGS-based strategies may allow significant improvement of the pre-treatment identification of high-risk patients, thus facilitating adequate dose-adjustment, and potentially improving patients' quality of life and reducing medical costs.

## Figures and Tables

**Table 1:** **Case series and case report**: *DPYD* gene sequencing in patients with severe fluoropyrimidine-related toxicity.

Analytical method	Cases description	Therapy	SNP Functional prediction	DPD phenotyping	Country (Ethnicity)	Main findings	Outcome	Ref
**CASE SERIES**								
Full gene sequencing (NGS)	**Case 1:** 65-year-old female with LARC. A few days after starting adjuvant treatment she experienced grade2-3 toxicities. CAPE was stopped and switched to mFOLFOX but the patient again developed early grade 3 toxicity that requiring hospitalization.	Neoadjuvant CAPE + RT. Adjuvant XELOX switched after toxicity to mFOLFOX-6	Literature search		Saudi Arabia (NS)	The patient was heterozygous for the rare *DPYD* variant rs371313778, c.2434G>A (p.Val812lle), which has normal activity *in vitro* with uncertain phenotypic significance. This variant has never before been identified in a case of toxicity.	FOLFOX was restarted with 5-FU at 50% dose reduction with further titration in subsequent cycles. The planned adjuvant treatment was completed.	29 30
Whole gene sequencing (NGS)	**Case 2**: 64-year-old female, a cousin of case 1, with stage III colon adenocarcinoma, sigmoid primary. Intermediate *DPYD* metabolizer.	Adjuvant XELOX, with CAPE at 40% dose-reduction based on family history of severe toxicity FL-related.	Literature search		Saudi Arabia (NS)	The patient was homozygous for the rare *DPYD* variant c.1601G>A (p.Ser534Asn, DPYD*4) previously linked to clinical DPD deficiency.	The reduced initial dose prevented the development of severe toxicity. CAPE dose was then increased by 5-10% but the patient developed toxicity requiring hospitalization. Therapy was continued with CAPE at 60% of standard dose.	
Whole gene sequencing (NGS)	**Case 3**: 66-year-old males with LARC. CAPE was stopped at week 4 of treatment due to severe toxicities requiring hospitalization.	Neoadjuvant CAPE + RT			Saudi Arabia (NS)	The patient was heterozygous for the very rare *DPYD* variant c.257C>T (p.Pro86Leu) previously linked to clinical DPD deficiency.	The patient concluded the planned therapy with only RT.	
MLPA and array-based comparative genomic hybridization analysis; All exons + flanking intronic regions + intron 10 sequencing (Sanger method)	8 pts (F, n=3; M, n=5) with GI cancers (62.5%, CRC; 25.5%, rectal cancer; 12.5%, esophageal cancer) and reduced DPD activity: 6 pts experienced early-onset grade 3-4 toxicity and 2 pts lethal grade 5 toxicity. One additional female patient with pre-treatment detected impaired DPD activity was treated with reduced FL dose without developing severe toxicity.	8 pts received standard dose of FL-containing regimen (UFT, n=1; 5-FU, n=1; CAPE, n=6) ± RT. One patient received a CAPE dose reduced by 50%.	RNA (cDNA) sequencing; Functional analysis of recombinantly-expressed DPD mutants; Crystal DPD structure analysis	DPD enzyme activity assay on PBMCs	Dutch (n=8); Danish (n=1) pts (Caucasian)	All 9 pts possessed a strongly reduced DPD activity (9-53%). A total of 21 *DPYD* aberrations, including 3 novel (c.1740+2T>C [p.Ser509_Lys580del], c.2407_2427del [p.Leu803_Gly809del], c.2843T>C [p.Ile948Thr]) and 4 very rare (c.321+1G>A [p.Cys79Thrfs*8], c.851G>T [p.Gly284Val], c.1280T>C [p.Val427Ala], c.2766+87G>A) were detected. All but one of these variants (c.2766+87G>A) were reported to have a functional impact on DPD activity. A novel aberration, the c.851_1524dup aberrant protein, was also detected.	--	46
Exon sequencing (Sanger method) in case of aberrant dHPLC pattern	3 female pts with CRC experiencing early-onset grade≥3 toxicity. Negative for DPYD*2A and c.2846A>T.	**Pts1**: 5-FU + LV + IRI + BV; **Pts2**: 5-FU + FA + OXA **Pts3**: adjuvant CAPE + OXA	Literature search; DPD structure analysis		Italy (NS)	3 novel non-synonymous *DPYD* variants (c.2509-2510insC [Leu -> Pro], c.1801G>C [Gly-> Ser], c.680G>A [Ser -> Gly]) were detected and possibly associated with a poor-metabolizer phenotype. These novel mutations could represent a detrimental variant and increase the effect of other SNPs identified in the same patient.	Treatment was discontinued / started with FL dose reduction.	31
Exon 14 RFLP and DNA sequencing (Sanger method)	**Case 1**: 76-year-old woman with colon cancer experiencing grade 3-4 toxicity and hospitalization after the third cycle.	Adjuvant 5-FU + Calcium folinate	Literature search; DPD structure analysis		Sweden (NS)	The patient was heterozygous for the functionally-relevant rare *DPYD* variant c.1796T>C (p.Met599Thr).	5-FU was reintroduced with a 50% dose reduction, without severe toxicity.	41
	**Case 2**: 75-year-old woman with colon cancer experiencing grade 2-3 toxicity after two thirds of the treatment	Adjuvant 5-FU + FA	RNA (cDNA) sequencing		Sweden (NS)	The patient was heterozygous for the *DPYD* splice site c.IVS14+ 17A>G variant which, however, does not appear to influence the splicing process and thus explains the observed toxicity.	Treatment was interrupted.	
Full coding region screening by dHPLC. Data validation by sequencing	4 patients with breast (n=3) or colorectal (n=1) cancer experiencing early-onset grade 3-4 toxicity.	Adjuvant CMF (n=2) Neoadjuvant FEC (n=1) Adjuvant 5-FU +FA (n=1)	Crystal DPD structure analysis	DPD enzyme activity assay on PBMCs	Germany (Caucasian)	In addition to the known and more frequent variants (c.85T>C [p.Cys29Arg], c.496A>G [p.Met166Val], c.1601G>A [p.Ser534Asn], c.1627A>G [p.Ile543Val, DPYD*5A], c.1896T>C [p.Phe632Phe]) a novel mutation (e.g., c.775A>G [p.Lys259Glu]) was detected. Rare variants* DPYD* combinations (e.g., c.85T>C + c.496A>G + c.1601G>A + c.1627A>G or c.496A>G + c.1601G>A) were detected and potentially associated with reduced DPD activity.	--	35
**CASE REPORT**								
Whole-Genome sequencing (NGS). Data validation by Sanger sequencing	59-year-old female with metastatic colon cancer experiencing grade 4 life-threatening toxicity following the first cycle of treatment. Negative for DPYD*2A, *13, c.2846A>T, c.1129-5923T>G- HapB3.	Neoadjuvant CAPE + OXA + BV	In silico tools: *DPYD*-Varifier;	DPD Enzyme Activity Assay	India (Indian, South Asian)	The patient was heterozygous for the very rare *DPYD* missense variant (rs755416212, c.704G>A [p.Arg235Gln]), which is predicted to be deleterious and significantly reduce DPD activity by 88%.	The patient was started on mFOLFOX regimen with a 75% reduced dose of 5-FU and no toxicity was observed.	39
Exon 14 sequencing; cDNA coding sequence sequencing (Sanger method)	81-year-old female patient with metastatic breast cancer experiencing grade 3-4 toxicity requiring hospitalization and suspension of CAPE following the first cycle of treatment. Negative for DPYD*2A, *13, c.2846A>T, c.1236G>A-HapB3.	CAPE for metastatic progression	In silico tools: SIFT, MutationTaster, PolyPhen-2, PROVEAN, HSF system; SwissModel web tools; RNA (cDNA) sequencing and real-time quantitative PCR assays;	Determination of U, UH2 and UH2/U ratio;	France (NS)	A partial DPD deficiency was determined. A reduced *DPYD* mRNA levels was detected. A novel *DPYD* variant c.1903A>G [p.Asn635Asp] was identified in a heterozygous state. However, the deleteriousness of this variant is doubt. Two other already known variants (i.e., rs1801265, c.85T>C, [p.Cys29Arg]; rs2297595, c.496A>G, [p.Met166Val] with potential clinical significance were identified.	The re-introduction of CAPE at lower doses was associated with a recurrence of severe toxicity, so treatment was stopped and new therapies were adopted.	42
All exons sequencing (Sanger method)	79-year-old female with breast cancer experiencing life-threatening toxicity after 1 week of treatment. The CAPE was suspended. Negative for DPYD*2A, *13, c.2846A>T,	Second-line CT based on CAPE	In silico tools: HSF system; RNA (cDNA) sequencing;	Determination of U, UH2 and UH2/U ratio.	Spain (Caucasian)	A novel rare functionally-relevant c.2242+1G>T splicing variant was detected. This variant produces a shorter mRNA and protein, which leads to a non-functional DPD protein and could explain the severe toxicity.	The patient was no longer treated with FL.	34
All exons + flanking intronic regions sequencing (Sanger method) Copy number variation by MLPA analysis	59-year-old female patient with a sigmoid adenocarcinoma. A heterozygosity for the DPYD*2A variant and a complete DPD deficiency (i.e., DPD activity in PBMCs) were determined pre-treatment.	Adjuvant CAPE + OXA (CAPE started with dose at 0.8% of originally planned dose)	Literature search;	DPD activity in PBMCs	The Netherlands (NS)	The DPYD*1/*2A genotype did not explain the complete DPD deficiency. A novel amplification of *DPYD* exons 17 and 18 was detected and associated with the DPD deficiency.	The pre-emptive dose reduction has prevented the development of potentially life-threatening toxicity.	36
Exome sequencing (NGS)	49-year-old female patient with resected stage III carcinoma of sigmoid colon developing grade≥3 toxicity after the first cycle requiring hospitalization.	Adjuvant CAPE + OXA	In silico tools: HSF system		Hong Kong (NS)	The patient was heterozygous for the novel intronic c.321+2T>C variant, a pathogenic splicing variant resulting in a non-functional allele.	CT was restarted with FOLFOX and with a dose of 5-FU reduced by 30%. The dose was then titrated, and the patient tolerated the subsequent cycles.	44
All exons sequencing (Sanger method)	37-year-old female with metastatic breast cancer who experienced early onset severe toxicities requiring hospitalization.	Adjuvant CAPE + trastuzumab	Literature search; DPD structure analysis		Italy (Caucasian)	The patient was a heterozygous carrier of four variants: c.257C>T [p.Pro86Leu], c.496A>G [p.Met166Val], c.1850C>T [p.Thr617Met] and c.2194G>A [p.Val732Ile]. The novel missense variant c.1850C>T (p.Thr617Met) in combination with the rare c.257C>T (p.Pro86Leu) was probably responsible for the severe life-threatening toxicity. The two variants could be deleterious.	The patient died due to multiorgan failure.	32
Promoter region + coding exons sequencing (Sanger method)	63-year-old female patient with Lieberkühn adenocarcinoma developing lethal toxicity 8 days after the first infusion of 5-FU. Patient was routinely tested for the DPYD*2A, *13, c.2846A>T, c.464T>A.	Adjuvant FOLFOX	In silico tools: UMD-Predictor; Literature search	Determination of U, UH2 and UH2/U ratio	France (Caucasian)	The patient, with complete DPD deficiency, was heterozygous for two* DPYD* variants: a novel 8-bp duplication (c.168_175dupGAATAATT, [p.Phe59Ter]) and DPYD*13. The novel variation resulted in a stop codon (p.Phe59Ter) and was likely responsible for the lethal toxicity.	She died 17 days after 5-FU administration.	43
All exons + adjacent intronic regions sequencing (Sanger method). Data validation by Pyrosequencing.	73-year-old female with colon cancer developing lethal toxicity 7 days after the first cycle.	Adjuvant 5-FU + LV	Protein truncation test in bacterial expression vector.	Determination of U, UH2 and UH2/U ratio and 5-FU level	Spanish (NS)	The patients, with decreased DPD enzyme activity, was wild-type for 22 potentially relevant variants analyzed by targeted genotyping. The rare novel functionally-relevant variant c.464T>A (p.Leu155Ter) was identified by sequencing and was the potential cause of life- threatening toxicity.	She died 19 days after 5-FU administration.	40
All exons sequencing (Sanger method)	35-year-old male with advanced caecum cancer developing a multiple organ dysfunction 2 days after onset of CT requiring hospitalization.	Adjuvant 5-FU + FA	Literature search		Germany (NS)	The patient was carrier of the rare variant c.1601G>A (p.Ser534Asn, *4) in addition to the more frequent non-pathogenetic c.85T>C (p.Cys29Arg) and c.1627A>C (p.Ile543Val) variants. The c.1601G>A was detected together with an intronic mutation (c.IVS13+40G>A); reduced enzyme activity was consistently observed for both variants.	The toxic symptoms, which required intensive treatment, eventually led to a full recovery.	38
Coding exons sequencing (Sanger method)	42-year-old female with advanced ovarian carcinoma developing grade 4 toxicities 4 days after the first single injection of 5-FU.	Palliative 5-FU + LV treatment for symptomatic liver metastases	DPD structure analysis		The Netherlands (NS)	The patient was heterozygous for the novel variant c.61C>T (p.Arg21Ter) that determines non-functional protein without any residual activity. The patient was also heterozygous for the DPYD*2A variant. The two variants were probably located on different alleles, thus causing a complete DPD deficiency DPD and the rapid onset of the lethal toxicity.	She died 21 days after the first push of 5-FU.	45
All exons + + 5' and 3' UTRs + promoter region by dHPLC. Data validation by sequencing (Sanger method)	53-year-old female with LARC experiencing severe toxicity after day 1 of CT; on the third day the 5-FU was discontinued (case 3 in the study).	Neoadjuvant 5-FU + RT	Literature search	DPD enzyme activity measured in human PBM cells.	USA (Caucasian)	The patient was heterozygous for the novel c.545T>A (p.Met182Lys) variation in addition to the rare c.2329 G>T (p.Ala777Ser) and the more frequent DPYD*5 (c.1627A>G, [p.Ile543Val]). Since DPYD*5 has not been shown to lead to any change in DPD enzyme activity, the partial DPD deficiency observed in the patient is due to c.545T>A, and/or c.2329G>T.	The patient died 1 week after the start of the 5-FU treatment.	33
cDNA sequencing; RFLP	57-year-old female with breast cancer experiencing severe toxicity during 5-FU treatment	--	Expression analysis in *Escherichia coli*;	DPD enzyme activity measured in human PBM cells; Determination of pyrimidines and their derivates.	Japan (Japanese, East Asian)	DPD activity is significantly decreased. The patient was heterozygous for 3 novel mutations (c.62G>A [p.Arg21Gln, DPYD*12], c.1003G>T [p.Val335Leu, DPYD*11], c.1156G>T [p.Glu386Ter]). Analysis of the family genome revealed that p.Arg21Gln and p.Glu386Ter are located on the same allele and p.Val335Leu on the other allele. The p.Val335Leu and p.Glu386Ter resulted in a significant loss of enzymatic activity and no activity, respectively. p.Arg21Gln showed no effect on the functionality of the enzyme.	--	37

**Abbreviations:** 5-FU, 5-fluorouracil; BV, bevacizumab; CAPE, capecitabine; CMF, cyclophosphamide + methotrexate + 5-FU; CRC, colorectal cancer; CT, chemotherapy; dHPLC, denaturing high-performance liquid chromatography; DPD (*DPYD*), dihydropyrimidine dehydrogenase; FA, folinic acid; FEC, 5-FU + epirubicin + cyclophosphamide; FOLFOX, 5-FU + OXA + leucovorin; GI, gastrointestinal; HSF Human Splicing Finder, IRI, irinotecan; LARC, locally advanced rectal cancer; LV, leucovorin; mFOLFOX, modified FOLFOX; MLPA, multiplex ligation-dependent probe amplification; MAF, minor allele frequency; NGS, next generation sequencing; NS, not specified; OXA, oxaliplatin; PBMCs, a peripheral blood mononuclear cell; PROVEAN, Protein Variation Effect Analyzer; Pts, patients; RFLP, Restriction fragment length polymorphism; RT, radiotherapy; SIFT, Scale-Invariant Feature Transform; SNP, single nucleotide polymorphism; U, uracil; UFT, Tegafur/uracil; UH2, dihydrouracil; XELOX, capecitabine + oxaliplatin.

**Table 2:** Characteristics of studies investigating a strategy based on *DPYD* gene sequencing to improve the prediction of fluoropyrimidine-related toxicity risk.

Analytical method	Patients population	Gender	Therapy	SNP Functional prediction	DPD phenotyping	Country (Ethnicity)	Main findings	Ref
All exons + splice junctions + 5' and 3' UTRs + proximal promoter region sequencing (NGS). Data validation by Sanger sequencing.	213 cancer pts (63.8%, colon; 19.2% rectum; 17%, other cancers) Negative for the DPYD*2A, *13, c.2846A>T, c.1236G>A-HapB3. Cases (n=109): grade≥3 toxicity Controls (n=104): no toxicity	F (51.2%); M (48.8%)	5-FU: 85.5% CAPE: 14.5% Monotherapy (7.0%), Combined therapy (93.0%); Concomitant drug: OXA (38.5%); IRI (35.7%); others (18.8%).	In silico tools: APF, PredictSNP algorithm, LOFTEE, SpliceAI, MicroSNiPer, MirSNP database; Structural modeling	Determination of U, UH2 and UH2/U ratio	Italy (Caucasian)	Carriers of at least one rare missense *DPYD* variant (MAF<0.01) had an increased risk in the first cycle (OR:16.20; P=0.013) and during the entire course of CT (OR:11.06; P=0.025) of developing grade≥3 toxicity.	21
Coding exons + flanking intron regions sequencing (NGS)	301 cancer pts (68.1%, CRC; 23.3% stomach; 17%, other cancers). Cases (n=55): grade3-4 toxicity Controls (n=246): grade0-2 toxicity	F (40.5%); M (59.5%)	5-FU-based therapy Monotherapy (28.9%), Combined therapy (71.1%);	In silico tools: SIFT, Polyphen-2		Japan (Japanese, East Asian)	Carriers of at least one rare *DPYD* variant (MAF<0.01) causing loss of function *in silico* had an increased risk of developing grade≥3 toxicity in the first two cycles (P=0.003).	54
Exome sequencing (Sanger method)	33 cancer pts (27.3%, breast; 72.7%, digestive tract cancer) Negative for DPYD*2A, *13, c.2846A>T, c.1236G>A-HapB3. Cases (n=11): grade≥3 toxicity within the first two cycles Controls (n=22): no grade≥3	F (81.8%); M (18.2%)	CAPE (63.6%) 5-FU (36.4%) Monotherapy (42.4%), Combined therapy (57.6%)	Literature search; In silico tools: SpliceAI, RegSNPs-intron	Determination of U, UH2 and UH2/U ratio	Spain (NS)	The functionally-relevant rare rs367619008 (c.187A>G, [p.Lys63Glu]) and rs200643089 (c.2324T>G, [p.Leu775Trp]) variants, together with the more frequent rs76387818 (c.1084G>A, [p.Val362Ile]) variant, increased the percentage of explained toxicities from 20-30% (with the 4 recommended markers) to 38-48%. An intronic rare variant potentially pathogenic (rs944174134, c.322-63G>A) was also identified.	52
All exons + intron/exon boundaries sequencing (NGS)	94 cancer pts (81.9%, colon; 18.1%, other cancers)	F (42.5%); M (57.5%)	na	In silico tools: HSF system, redictSNP algorithm	5-FU degradation rate assay	Italy (NS)	Exon sequencing, with information also on rare variants (MAF<0.05), allowed to recognize an additional 22.5% of pts carrying variants possible cause of DPD deficiency (5-FU degradation rate assay) compared to screening only the recommended variants (DPYD*2A, *13, HapB3 and c.2846A>T [p.Asp949Val]), which identify only 20% of DPD deficiencies.	47
All exome + flanking intronic regions + 3'/ 5'UTR sequencing (NGS)	243 advanced breast cancer pts	F (100.0%)	CAPE Monotherapy (88.5%), Combined therapy (11.5%)	In silico tools: UMD-Predictor system, HSF	Determination of U, UH2 and UH2/U ratio	France (NS)	The inclusion of seven rare (MAF<1%) *in vitro* deleterious *DPYD* alleles, all mutually exclusive (*2A, *13, c.2846A>T [p.Asp949Val], c.1475C>T [p.Ser492Leu], c.1774C>T [p.Arg592Trp], c.1025A>G [p.Asp342Gly], c.300C>A [p.Phe100Leu]) improved the performance of the genotyping test compared to the inclusion of only the 3 consensual variants (*2A, *13, c.2846A>T) for both grade 3-4 (sensitivity 26.7%, PPV 72.7%, RR 7.6, P<0.001) and grade 4 toxicities (sensitivity 60%, PPV 27.3%, RR 31.4, P=0.001).	48
Whole sequencing of the coding exon and flanking intron regions (Sanger method)	41 cancer pts (17.1%, stomach; 17.1% rectum; 51.2% colon; 4.9%, breast; 9.7%, pancreas) Cases (n=27): grade≥ 3 toxicity within the first three cycles. Controls (n=14): grade ≤ 1 toxicity during at least eight treatment cycles. Negative for DPYD*2A, *13, c.2846A>T, c.1236G>A-HapB3.	F (51.2%); M (48.8%)	CAPE (61.0%) 5-FU (39.0%) Concomitant drug: OXA (n=31); IRI (n=4); targeted agents (n=10)	In silico tools: SIFT, Polyphen2 and ClinVar; mRNA expression levels analysis; RNA (cDNA) sequencing; Structural modeling; Literature Search	Determination of U concentration	Spain (NS)	A novel rare nonsense functionally relevant variant (c.2197insA [p.Thr733AsnfsTer14]) was identified and most likely associated with DPD deficiency and early severe toxicity. In the case group, a 3'UTR novel variant (c*159A>G) with possible functional impact was also detected and was a good candidate to explain the observed toxicity. Two other very rare variants (c.1218G>A [p.Met406Ile], c.2071G>T [p.Val691Leu]) were found in case group but both with controversial functional results.	53
Coding regions sequencing (NGS) KASPar technology for genotyping all study population.	968 cancer pts (89.0%, colon; 11.0% rectum) 'HiTox' (n=100): early-onset grade 3-4 'LoTox'(n=100): no toxicity	F (43.0%); M (57.0%)	CAPE ± BV	In silico tools: SIFT, Polyphen, PhyloP, MutationTaster; Literature search		United Kingdom (Caucasian)	HiTox and LoTox groups were sequenced and a rare *DPYD* missense variant (c.1651G>A; [p.Ala551Thr]) was identified in the Hitox group. This variant was found in only one of the 968 pts who experienced grade 4 hematological toxicity. The variant was predicted to be strongly damaging and was reported to be causal for toxicity.	50
All exons + intronic neighborhood regions sequencing (Sanger method)	28 cancer pts (57.1%, colon; 17.9% rectum; 21.4% breast; 3.6% stomach) developing grade≥3 within the first three cycles. Negative for the DPYD*2A, *13, c.2846A>T, c.1236G>A-HapB3	F (57.1%); M (42.9%)	5-FU: 35.7% CAPE: 64.3% Monotherapy (42.9%), Combined therapy (57.1%); Concomitant drug: OXA (39.3%); IRI (10.7%); others (14.3%).	In silico tools: SIFT, PolyPhen-2, *DPYD*-verifier, HSF system; Structural modeling	Determination of U, UH2 and UH2/U ratio	Spain (NS)	Description and functionally characterization of the common and rare variants, including two very rare mutations (c.2087G>A, [p.Arg696His] and the functionally-relevant c.2324T>G, [p.Leu775Trp]). 25 (25/28, 90%) pts had a least 1 variant in *DPYD* coding sequence, and about half of them were potentially deleterious.	49
Coding regions sequencing (Sanger method)	15 cancer pts with DPD deficiency and developing severe toxicity within the first or second cycle. Pts were tested for the DPYD*2A, *13, c.2846A>T, c.464T>A.		5-FU or CAPE	In silico tools: UMD-Predictor; Literature search	Determination of U, UH2 and UH2/U ratio	France (Caucasian)	In addition to the three tested variants, some rare/ novel deleterious *DPYD* missense variants (c.257C>T [p.Pro86Leu], c.623G>A [p.Arg208Gln], c.1027A>C [p.Thr343Pro]), not included in the genotyping screening method, were also detected.	43
All exons + exon/intron boundaries sequencing (Sanger method) dHPLC and MALDI-TOF-based assays for genotyping all study population.	683 pts with GI or breast cancer. 573 pts developed grade 0-2 toxicity; 110 pts developed grade 3-4 toxicity)**. Pts were tested for the DPYD*2A **All 28 pts with grade 4 and 28 pts with grade 3 toxicity as well as 28 control patients (grade 0-2) were sequenced. Genetic variants apparently associated with grade 3-4 toxicity were then genotyped in a larger population.	F (43.9%); M (56.1%)	5-FU ± FA or levamisole	In silico tools: PolyPhen		Germany (NS)	In addition to DPYD*2A, a total of 12 further exonic mutations were identified including four novel variants (c.623G>A [Arg208Gln], c.775A>G[p.Lys259Glu], c.1391T>C [p.Val464Ala], c.2858G>C [p.Cys953Ser]). The novel functionally-relevant variant c.2858G>C was identified in one patient with grade 4 mucositis and was not detected in any other patient.	51

**Abbreviations**: 5-FU, 5-fluorouracil; APF, ADME-optimized Prediction Framework; BV, bevacizumab; CAPE, capecitabine; CRC, colorectal cancer; CT, chemotherapy; dHPLC, denaturing high-performance liquid chromatography; DPD (*DPYD*), dihydropyrimidine dehydrogenase; HSF, Human Splicing Finder, IRI, irinotecan; LOFTEE, Loss-Of-Function Transcript Effect Estimator; MAF, minor allele frequency; NGS, next generation sequencing; NS, not specified; OXA, oxaliplatin; PPV, positive predictive value; Pts, patients; RR, relative risk; SIFT, Scale-Invariant Feature Transform; SNP, single nucleotide polymorphism; U, uracil; UH2, dihydrouracil; UTR, untranslated region.

**Table 3:** List of the rare and novel *DPYD* variants potentially associated with fluoropyrimidine-related toxicity that have been reported in published case reports, case series and clinical studies selected for this review.

*DPYD* variant^§^		Typology	Rs ID	Classification by authors#	Variant Allele Frequency^&^						Ref
					All	African (AFR)	American (AMR)	East Asian (EAS)	European (EUR/NFE)	South Asian (SAS)	
NM_000110.4:c.61C>T	NP_000101.2:p.Arg21Ter	Stop-gained	rs72549310*	Novel	<0.001	<0.001	0	0	<0.001	0	45
NM_000110.4:c.62G>A	NP_000101.2:p.Arg21Gln, DPYD*12	Missense	rs80081766*	Novel	<0.001	0	0	0	<0.001	<0.001	37
NM_000110.3:c.168_175dupGAATAATT	NP_000101.2:p.Phe59Ter	Stop-gained	NA	Novel							43
NM_000110.4:c.187A>G	NP_000101.2:p.Lys63Glu	Missense	rs367619008	Rare	<0.001	0	0	<0.001	<0.001	0	52
c.2197insA (sequence aligned with NC_000001.11 GRCh38.p13)	p.Thr733AsnfsTer14	Stop-gained	NA	Novel							53
NM_000110.4:c.257C>T	NP_000101.2:p.Pro86Leu	Missense	rs568132506	Rare/Very Rare	<0.001	0	<0.001	0	<0.001	<0.001	43 29 32
NM_000110.4 c.300C>A	NP_000101.2:p.Phe100Leu	Missense	NA	Rare							48
NM_000110.3 c.321+2T>C		Splice-site	NA	Novel							44
NM_000110.4:c.321+1G>A	NP_000101.2:p.Cys79Thrfs*8	Splice-site	rs746368304	Rare	<0.001	0	0	0	<0.001	0	46
NM_000110.4:c.464T>A	NP_000101.2:p.Leu155Ter	Stop-gained	rs2101026231*	Novel	na	na	na	na	na	na	40
NM_000110.4:c.545T>A	NP_000101.2:p.Met182Lys	Missense	rs779728902*	Novel	<0.001	0	<0.001	0	<0.001	0	33
NM_000110.4:c.623G>A	NP_000101.2:c.Arg208Gln	Missense	rs376073289*	Novel/Rare	<0.001	0	0	<0.001	<0.001	0	43 51
c.680G>A	Ser -> Gly	Missense	NA	Novel							31
NM_000110.4:c.704G>A	NP_000101.2:p.Arg235Gln	Missense	rs755416212	Very Rare	<0.001	0	0	0	<0.001	0	39
NM_000110.4:c.775A>G	NP_000101.2:p.Lys259Glu	Missense	rs45589337*	Novel	0.006	0.001	0.003	0	0.008	0.005	35 51
NM_000110.4:c.851G>T	NP_000101.2:p.Gly284Val	Missense	rs777220476	Rare	<0.001	0	<0.001	0	<0.001	0	46
NM_000110.4:c.1003G>T	NP_000101.2:p.Val335Leu, DPYD*11	Missense	rs72549306*	Novel	<0.001	<0.001	<0.001	<0.001	0	0	37
NM_000110.4:c.1025A>G	NP_000101.2:p.Asp342Gly	Missense	rs769709846	Rare	<0.001	0	0	0	<0.001	0	48
NM_000110.3:c.1027A>C	NP_000101.2:p.Thr343Pro	Missense	NA	Novel							43
NM_000110.4:c.1156G>T	NP_000101.2:p.Glu386Ter	Stop-gained	rs78060119*	Novel	<0.001	0	0	0	<0.001	<0.001	37
NM_000110.4:c.1218G>A	NP_000101.2:p.Met406Ile	Missense	rs61622928	Very rare	0.005	0.065	0.004	0	<0.001	<0.001	53
NM_000110.4:c.1280T>C	NP_000101.2:p.Val427Ala	Missense	rs200693895	Rare	<0.001	0	0	0	<0.001	<0.001	46
NM_000110.4:c.1391T>C	NP_000101.2:p.Val464Ala	Missense	rs370707404*	Novel	<0.001	<0.001	0	0	<0.001	0	51
NM_000110.4:c.1475C>T	NP_000101.2:p.Ser492Leu	Missense	rs72549304	Rare	<0.001	<0.001	0	0	<0.001	<0.001	48
NM_000110.4:c.1601G>A	NP_000101.2:p.Ser534Asn, DPYD*4	Missense	rs1801158	Rare	0.015	0.004	0.011	<0.001	0.020	0.009	29 38 35
NM_000110.4:c.1651G>A	NP_000101.2:p.Ala551Thr	Missense	rs777425216	Rare	<0.001	<0.001	0	0	<0.001	0	50
NM_000110.3:c.1740+2T>C	NP_000101.2:p.Ser509_Lys580del	Splice-site	NA	Novel							46
NM_000110.4:c.1774C>T	NP_000101.2:p.Arg592Trp	Missense	rs59086055	Rare	<0.001	<0.001	0	0.002	<0.001	<0.001	48
NM_000110.4:c.1796T>C	NP_000101.2:p.Met599Thr	Missense	rs147601618	Rare	<0.001	<0.001	0	0	<0.001	0	41
c.1801G>C	Gly-> Ser	Missense	NA	Novel							31
NM_020442.6:c.1850C>T	NP_065175.4:p.Thr617Met	Missense	rs367837827*	Novel	<0.001	0	0	0.002	<0.001	<0.001	32
c.1903A>G (RefSeq: NM_000110, Transcript ID: ENST00000370192.7)	p.Asn635Asp	Missense	NA	Novel							42
NM_000110.4:c.2071G>T	NP_000101.2:p.Val691Leu	Missense	rs202212118	Very rare	<0.001	0	<0.001	0	<0.001	0	53
NM_000289.6:c.2087G>A	NP_000280.1:p.Arg696His	Missense	rs41291971	Very rare	0.010	0.003	0.006	0.002	0.016	0.002	49
NM_000110.4:c.2242+1G>T		Splice-site	NA	Novel							34
NM_000110.4:c.2324T>G	NP_000101.2:p.Leu775Trp	Missense	rs200643089	Rare/Very rare	<0.001	0	<0.001	0	<0.001	0	52 49
NM_000110.4:c.2329G>T	NP_000101.2:p.Ala777Ser	Missense	rs672601276	Rare	<0.001	0	<0.001	0	<0.001	0	33
NM_000110.3c.2407_2427del	NP_000101.2:p.Leu803_Gly809del	Deletion	NA	Novel							46
NM_000110.4:c.2434G>A	NP_000101.2:p.Val812lle	Missense	rs371313778	Rare	<0.001	0.001	<0.001	<0.001	<0.001	<0.001	30
c.2509-2510insC	Leu -> Pro	Missense	NA	Novel							31
NM_000110.4:c.2766+87G>A		Intronic	rs556768807	Rare	<0.001	0	0	0	<0.001	0	46
NM_000110.4:c.2843T>C	NP_000101.2:p.Ile948Thr	Missense	NA	Novel							46
c.2858G>C (NC_000001.8 was used as reference sequence)	Cys953Ser	Missense	NA	Novel							51
IVS14+17A>G		Splice-site	NA	NA							41
c*159A>G (sequence aligned with NC_000001.11 GRCh38.p13)		3'UTR	NA	Novel							53
Exons 17 and 18 (Ref Seq NM_000110.3; Ensembl ENST00000370192)		amplification		Novel							36

**Abbreviations:** NA, not available; UTR, untranslated region ^§^ When the information about a transcript was not available, the variation change was given as reported by the authors in the original paper. ^&^Frequency data obtained by Ensembl 51 from gnomADe database or, when the data are not available, from 1000 Genomes European population database. # The classification as “rare”, “very rare”, or “novel” (i.e., absent from available public database) provided according to the authors of the studies, and based on the information available at the time of the original paper publication. *The variant was classified as novel at the time of publication of the original paper; an rs ID was assigned only afterwards.

## Abbreviations

5-FU: 5-fluorouracil
APF: ADME-optimized prediction framework
DPD, DPYD: dihydropyrimidine dehydrogenase
DPYS: dihydropyrimidinase
FL: fluoropyrimidine
MAF: minor allele frequency
U: uracil
UH_2_: dihydrouracil
